# Information-Driven Design as a Potential Approach for 3D Printing of Skeletal Muscle Biomimetic Scaffolds

**DOI:** 10.3390/nano10101986

**Published:** 2020-10-08

**Authors:** Silvia Baiguera, Costantino Del Gaudio, Felicia Carotenuto, Paolo Di Nardo, Laura Teodori

**Affiliations:** 1 Departmet of Fusion and Technologies for Nuclear Safety and Security, Diagnostic and Metrology (FSN-TECFIS-DIM), ENEA, 00196 Roma, Italy; carotenuto@med.uniroma2.it; 2 Department of Clinical Science and Translational Medicine, University of Rome “Tor Vergata”, 00133 Roma, Italy; dinardo@uniroma2.it; 3 Interdepartmental Center of Regenerative Medicine (CIMER), University of Rome “Tor Vergata”, 00133 Roma, Italy; 4 E. Amaldi Foundation, Via del Politecnico snc, 00133 Rome, Italy; costantino.delgaudio@fondazioneamaldi.it; 5 L.L. Levshin Institute of Cluster Oncology, I. M. Sechenov First Medical University, 119991 Moscow, Russia

**Keywords:** decellularized extracellular matrix, engineered muscular construct, stereolithography, volumetric muscle loss

## Abstract

Severe muscle injuries are a real clinical issue that still needs to be successfully addressed. Tissue engineering can represent a potential approach for this aim, but effective healing solutions have not been developed yet. In this regard, novel experimental protocols tailored to a biomimetic approach can thus be defined by properly systematizing the findings acquired so far in the biomaterials and scaffold manufacturing fields. In order to plan a more comprehensive strategy, the extracellular matrix (ECM), with its properties stimulating neomyogenesis and vascularization, should be considered as a valuable biomaterial to be used to fabricate the tissue-specific three-dimensional structure of interest. The skeletal muscle decellularized ECM can be processed and printed, e.g., by means of stereolithography, to prepare bioactive and biomimetic 3D scaffolds, including both biochemical and topographical features specifically oriented to skeletal muscle regenerative applications. This paper aims to focus on the skeletal muscle tissue engineering sector, suggesting a possible approach to develop instructive scaffolds for a guided healing process.

## 1. Introduction: the Current Scenario

Severe muscle injuries (muscle loss greater than 20%) are beyond the endogenous regenerative capacity of skeletal muscle (SM) tissue, and the current gold standard approach (autologous tissue transfer) is often associated with donor site morbidity, functional deficiency and graft failure [1,2]. Therefore, alternative and effective treatments, such as engineered muscle tissues, become a necessity.

Tissue engineering has usually been considered a promising applicative option to address urgent and critical clinical needs. Moreover, systems biology techniques can further support the collection of the desired output by developing physiologically relevant bio-engineered tissues [3]. The potential of these multidisciplinary approaches can pave the way to the definition of novel and original medical and surgical protocols capable of overcoming most of the drawbacks elicited by conventional therapies, such as functional impairment, reduced movement ability, severe immune response risks and high costs [1,2]. Such an expectation has driven almost all the investigations published so far aimed to design suitable scaffolds to actually promote muscular regeneration, closely mimicking the morphological, biochemical and functional properties of the specific natural extracellular matrix (ECM) [4,5,6]. This is a keypoint of the classic tissue engineering paradigm in which the scaffold acts as a temporary ECM, providing all the instructive cues guiding the formation of the autologous tissue, and is capable of remodeling according to the physiological processes of the host. Clearly, this outcome should avoid the typical limitations related to tissue/organ transplantation and conventional implantable prostheses, such as the lack of growth and integration [7,8].

In this regard, the challenge of tissue engineering is highly demanding, and the design of ad hoc scaffolds has to be accurately performed in order to offer a valid alternative. In particular, SM comprises bundles of long, highly aligned, striated, multinucleated fibers organized in a hierarchical manner within the ECM. Thus, a parallel-aligned structure, which implies dealing with an anisotropic tissue that contributes to influencing cellular orientation and maturation, should provide an overall replication of the physiologic microenvironment, as this is pivotal when seeking to develop an engineered biomimetic muscle. Thus, the selection of both peculiar materials and fabrication techniques that support the realization of a three-dimensional microarchitecture similar to that of the SM ECM plays a major role is providing functional engineered muscle tissues. However, these starting design considerations are just a partial solution to a large-scale problem. It is well-known that microstructure, stiffness and porosity, to mention only the most common and controllable features, represent the first input for dictating cell response. However, it is also known that this condition may be insufficient to promote the desired biological outcome. Dealing with a comprehensive tissue engineering protocol underlines, therefore, the necessity of developing a biomimetic approach to preparing an integrated materials/cells/signaling construct that orchestrates a univocal result. For this aim, several proposals targeted at muscle regeneration have already been presented, focusing on (i) the materials, synthetic and natural ones, and their combinations [5,6,9], (ii) the fabrication techniques to evaluate their role on scaffold morphology and properties [10,11,12], (iii) the surface functionalization [13], (iv) the cells to be used, stem cells or already committed [6,10], (v) the addition of different nanomaterials to deal with constructs responsive to electrical, magnetic and photothermal stimulation [14] and (vi) the methods to specifically induce vascularization, innervation and contractility [10,13]. More than interesting and promising findings have been reported; however, the proper cellular microenvironment to bioengineer the SM construct has not, to date, been found, and there is still a clinical need to introduce new strategies that can facilitate safe and large muscle tissue repair and regeneration [13]. This work, therefore, proposes a possible technical/functional approach for designing and preparing suitable biomimetic scaffolds for SM regeneration, starting from the experimental findings collected so far and focusing on decellularized ECM to be used as a starting biomaterial for bioprinting and stereolithography.

## 2. Moving Towards Biomimetic Engineered Muscular Constructs

Biomimetic scaffolds, usually recalled as potential three-dimensional structures for an effective healing protocol, should be accurately designed to replicate one or more attributes of a biological environment produced by a living organism. This expected outcome involves several key parameters that need to be critically assessed and selected since it is unlikely that all the ECM characteristics can be reproduced. Biomaterials are pivotal, as their interaction with the seeded cells will determine the final result of the tissue-engineered construct. For this aim, dealing with biologically derived materials seems to be a reasonable option, if the most relevant biochemical cues are preserved [9,15,16].

Noteworthy, additive manufacturing represents a recent generation of appealing means to develop bioactive scaffolds, largely recapitulating biochemical and functional features of the tissue to be healed. However, for most of the 3D printing technologies, the resolution that can be achieved is not comparable to the characteristic dimensions of the structural elements of the ECM to be resembled, and this may impair the desired result related to the natural microarchitecture. In this context, stereolithography could instead usefully support the fabrication of biomimetic scaffolds for muscle repair, as it is characterized by a higher resolution level (the highest among 3D printing techniques for tissue engineering), and thus capable of fabricating aligned structural elements with the muscle fiber characteristic size. This approach represents a promising scenario if the processing solution components are properly selected. Decellularized ECM (dECM) can be a suitable choice to address this issue, as it can be modified to act as a photopolymerizable hydrogel, intrinsically providing a number of biological cues to guide cell response. Herein, after an overview of dECMs used as bioinks and in stereolithographic setups, a design-oriented process, starting from histological images and SM dECM applications, is suggested in order to propose biomimetic SM dECM-based scaffolds for muscular regeneration.

### 2.1. SM dECM-Based Hydrogel: A Material for Biomimetic Engineered Muscular Constructs?

The bioink is a printable hydrogel containing nutrients, ECM components and cells in various permutations that can be crosslinked in order to acquire stability and tailored structural properties. This allows dealing with a three-dimensionally seeded scaffold, which is one of the goals to be reached for a real tissue-engineered scaffold. An ideal bioink should (i) provide tissue-specific biological, physical and mechanical cues, (ii) be biocompatible and (iii) have good printability and biodegradability [17]. Possible limitations for the fabrication process can be related to the rheological characteristics of the hydrogel, the shear stress values experienced by cells during the process and the photopolymerization needed to crosslink the scaffold, which in the end may negatively affect cell viability [12].

Different and specific bioinks, both natural and synthetic, have already been considered for bioprinting SM constructs [12,18]. Synthetic bioinks are characterized by tunable mechanical properties and crosslinking capacity, but also by insufficient cellular adhesion. In contrast, natural bioinks provide biological cues but have poor mechanical properties [19]. Moreover, a large number of them could not actually represent the complexity of natural tissues and thus are inadequate to recreate a suitable microenvironment for cell engraftment, survival and function. dECM, retaining specific tissue components, including collagen, glycosaminoglycans, growth and differentiation factors, can recapitulate most of the features of natural ECM, if not all. Thus, dealing with bioinks made of only dECM, providing cells with their natural microenvironment, could represent a further step towards the design of a real tissue-mimicking scaffold. In this context, dECM bioinks have been derived from different organs [17,20,21]. The obtainment of dECM-based bioinks for 3D printing involves different steps [17,22,23]. The first is the decellularization of the tissue of interest by removing the cells while preserving the ECM. Several physical, chemical, enzymatic and biological processes can be adopted; the selection depends on several factors, such as tissue and organ thickness, density and lipid content [20,21]. To prepare a printable material, dECM needs to be processed as a hydrogel: ECM is usually crushed into a powder form and solubilized in a physiological buffer solution (generally, enzymatic acidic digestion with a pH adjusted to accommodate cells, if to be included in the final formulation) in order to deal with a solution at 10 °C and a gel at 37 °C. dECM gels may have the intrinsic mechanical strength to be directly printed or need to be mixed with a crosslinking agent, reacting during the printing process [17,20]. Moreover, a carrier polymer can also be used to increase the solubility, tune the viscosity, or induce/enhance the post-cross-linking of the bioink [17,22].

Focusing on engineered SM constructs, recent studies based on additive manufacturing approaches using SM dECM as a bioink have shown promising results. Choi et al. demonstrated that an SM dECM bioink had sufficient printability for producing different shapes of 3D freestanding volumetric muscle constructs, indicating that this bioink can be usefully considered to design and fabricate the original structures of injured muscles prior to implantation [24]. Moreover, cells were encapsulated in a microenvironment similar to that of the native ECM, offering an additional advantage for myogenesis [24]. The same group demonstrated that prevascularized 3D printed muscle constructs co-axially fabricated with muscle and vascular dECM bioinks successfully mimicked the hierarchical architecture of vascularized muscles with improved de novo fiber formation, vascularization, innervations and 85% functional recovery in volumetric muscle loss injuries [25]. SM dECM has also been chemically modified to impart photo-crosslinkable properties and thus to enhance mechanical stability. By employing a multi-crosslinking approach, Skardal et al. achieved a soft, extrudable material for bioprinting, which could be further stabilized after deposition in order to reach the elastic modulus of a targeted material and thus match that of the tissue of interest [26]. Recently, Kim et al. chemically modified SM dECM and fabricated a uniaxially aligned/micro-topographical SM structure, suggesting that this construct may lead to efficient functional recovery of damaged or diseased SM tissues [27]. Indeed, the biochemical cues given by SM dECM accelerated myogenic differentiation, while the topographical ones allowed achieving a suitable cellular orientation [27].

These 3D printed tissue models represent a critical proof of concept for the use of dECMs as a proper bioink to prepare engineered muscular constructs. Moreover, it has been demonstrated that ECM degradation releases growth factors (such as basic fibroblast growth factor and vascular endothelial growth factor) and low molecular weight matricryptic oligopeptides to recruit and influence stem or endogenous progenitor cells that play a role in the constructive and functional remodeling process, including vasculature and innervation formation [28,29].

Although further studies are needed, the bioactive features of natural materials can support the generation of an innovative class of implantable devices capable of recapitulating the main functional characteristics of the tissue to be healed. However, this is a part of the requested solution, as ad hoc manufacturing methods should be selected or developed in order to correctly deal with the desired scaffold.

### 2.2. Stereolithography: A Suitable Fabrication Technique for Engineered Muscular Constructs?

Stereolithography is a 3D printing technique based on the layer photopolymerization of specific materials by means of light irradiation, usually UV, leading to a final stacked scaffold as the build stage is vertically translated. This fabrication method is characterized by an intrinsic high resolution, the highest among all the additive manufacturing techniques (about 6 μm), which allows finely controlling external geometry and internal architecture of the designed scaffold to realize complex geometries [30]. In addition, a reliable modification can be further considered to include additional functional cues [31].

Several working principles can be implemented, acting, for instance, on the computer-controlled movement of the light source to polymerize each layer of the structure, or to directly polymerize the whole layer by means of an array of thousands of micro-mirrors reflecting light in a predefined spatial pattern, i.e., the digital micro-mirror device [32].

The intrinsic high resolution can be further improved by two-photon stereolithography, reaching about 200 nm, by focusing two consecutive photons within the focal volume of a laser beam, typically less than 1 µm^3^ [33]. This experimental approach can allow the fabrication of scaffolds with nanoscale features similar to those of natural ECM, thus leading to assessing cell behavior in this kind of environment [34].

To overcome stereolithography limitations, such as the necessity to use photopolymerizable solutions containing potentially cytotoxic UV-activated photo-initiators, different chemical and biological functional agents have been conjugated to dECM [26,27,35,36]. The collected results suggested that these photo-crosslinking molecules have minimal negative effects on cell viability and function [26,27,35,36]. In particular, vitamin B2, activated by long-wavelength UV radiation, showed to be non-toxic, significantly increased tissue rigidity and provided therapeutic outputs, proving that it is a completely biocompatible photo-crosslinking agent [37,38]. Moreover, it has been recently demonstrated that the vitamin B2-UVA crosslinking approach is an effective strategy to enhance ECM-hydrogel performance for tissue engineering and regenerative medicine applications, without inducing significant effects on cell viability and apoptosis, and therefore, being a potentially attractive and tunable means for scaffolding strategies [39,40,41]. To date, muscle-derived dECM has not yet been assessed for the preparation of biomimetic scaffolds by means of stereolithography. According to the findings already reported and including tissue-specific ECM, a possible strategy could then be developed in order to support the definition of ad hoc therapeutic protocols for large muscle defects.

## 3. Proposal of a Biomimetic Scaffold for the Treatment of Severe Muscle Injuries

**Figure 1:**
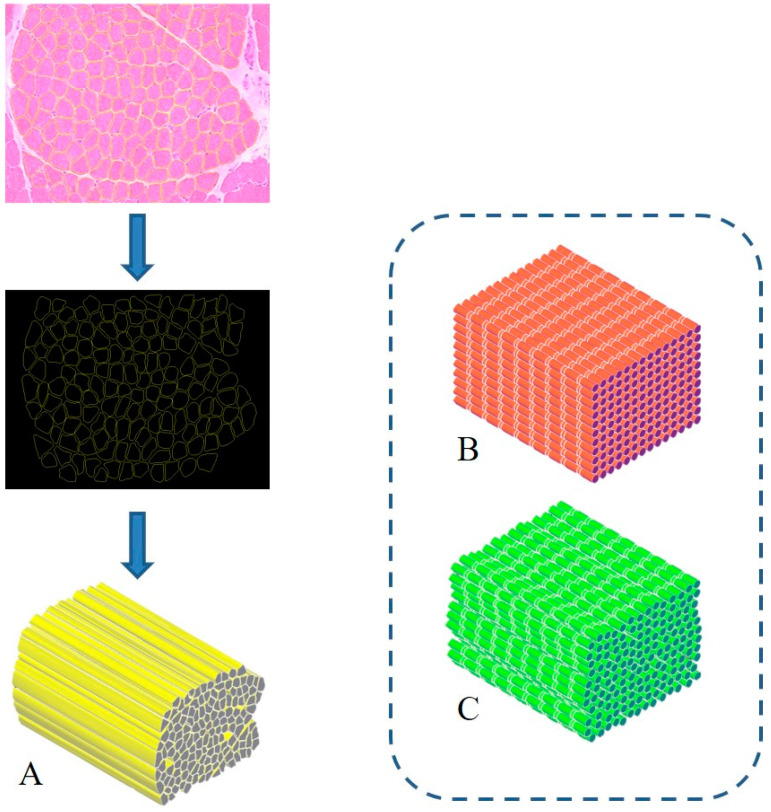
Biomimetic design of scaffold for skeletal muscle (SM) tissue engineering. A three-dimensional bioactive construct can be fabricated thanks to stereolithography, resembling the muscle structure and including SM decellularized extracellular matrix (dECM) (**A**). Similarly, a simple (**B**) and a more realistic (**C**) control case can be developed, mimicking the striated structure.

The treatment of severe muscle injuries can be an investigational field in which the synergic potential of dECM and stereolithography might support the development of an effective biomimetic scaffold. Indeed, stereolithography could close the loop, allowing the fabrication of a scaffold with the characteristic dimensions, alignment and orientation of the muscle fibers. A similar approach can pave the way to the development of scaffolds substantially made of the tissue-specific “biomaterial”. Moreover, it can be assumed that such a solution could be characterized by extreme biocompatibility and valuable matching properties with the biological environment. Furthermore, with the use of physiological datasets (histological images, SEM micrographs or medical images) and computer-aided design (CAD) to model scaffold fabrication, stereolithography can allow creating scaffolds with precise tissue architectures [42]. The output of medical imaging can effectively support the accurate definition of a scaffold resembling the target tissue in order to provide, e.g., stability and flow transport as peculiar properties of the extracellular microenvironment. The integration of an information-driven design and CAD-based micromanufacturing approach has been previously highlighted, reviewing a number of solutions from different fabrication techniques, always referring to the ECM characteristics to be reproduced [43]. This approach, for instance, was considered for preparing 3D printed devices for neural regeneration starting from magnetic resonance imaging, computed tomography, and/or 3D virtual visualization to acquire topological data for the tailored production of structures that match the injured microenvironment, a promising methodology for patient-specific implants [44]. Similar considerations can be presented regarding the orthopedic field in which imaging data can pave the way for personalized treatment [45]. However, it should be underlined that most of the solutions proposed so far are generally focused on large defects treated with scaffolds that may not finely reproduce the ultrastructure of the tissue to be healed. In this regard, the use of morphological images with micro/nano details of the tissue of interest (such as histological images and/or SEM micrographs) [46] could allow the development of biomimetic structures, resembling the natural ECM and can actually provide a step forward in the definition of novel therapeutic protocols. In this regard, the design-oriented process implementing stereolithography can start, for instance, from histological images or scanning electron micrographs of muscle fibers to be considered as the architectural structure to be 3D printed. Following this suggestion, Figure 1 shows the planning approach to prepare a scaffold aimed at resembling the fiber morphology and packing density of an SM structure. In this regard, the proposal elaborates histological observations to design aligned fibers reproducing the muscle cross-section. Moreover, considering that SM fibers appear striated due to the sarcomeres alignment, two other scaffolds could be considered: a scaffold composed of organized parallel, aligned and striated fibers (as control) (Figure 1B) and a scaffold composed of striated fibers organized as in the SM structure (Figure 1C). These latter options can represent a simplified version of the SM scaffold, which can further support the optimization of the design process towards the final applicative proposal.

In addition, to complete the design stage, computational models can be proposed as a means to investigate and predict either the fluid dynamics and the mechanical response in a simulated relevant environment, e.g., a bioreactor or physiological conditions. However, several requirements should be accomplished in order to deal with a realistic solution in terms of, for instance, experienced shear stress, mass transport phenomena and local stiffness properties. This implies an accurate validation step, which depends on the implementation of biological data as input and numerical physiological matching output, supported by high-performance computing resources.

Such an approach can lead to an effective tissue-engineered construct combining most of the requirements needed for a substantial biomimetic investigation. The possibility of resembling the tissue microarchitecture according to physiological datasets, of modeling the printing input to be fabricated by means of tissue-specific biomaterials and ad hoc 3D printing techniques capable of assuring the scaffold resolution can then actually allow to mimic SM tissue and address the crucial issue of severe muscle injuries. More refined solutions can obviously be considered, but the different manufacturing alternatives within the stereolithography sector can usefully and promptly realize this kind of substrate. Hydrogel formulation, light source type and power, exposure time and patterning strategy concur to modulate the final mechanical characteristics to match those of the physiological site of implantation.

Therefore, a rational and comprehensive plan should be developed that includes and further exploits all the keypoints here addressed: (i) the selection of the material(s) to formulate the hydrogel to be processed, combining dECM, cells and possibly other polymers; (ii) the CAD input for the stereolithography setup, taking advantage of the resolution and accuracy of the technique that can actually allow collecting scaffolds morphologically and functionally similar to the natural ECM; and (iii) the implementation of the most suitable fabrication methodology, e.g., single- or two-photon stereolithography.
